# Effects of different rehydration temperatures on rehydration and nutritional quality of dried daylily

**DOI:** 10.7717/peerj.20408

**Published:** 2025-12-16

**Authors:** Pei-Zhuo Liu, Ya-Hui Wang, De-Bao Xu, Jing-Wen Li, Jie-Xia Liu, Li-Xiang Wang, Ai-Sheng Xiong

**Affiliations:** 1 State Key Laboratory of Crop Genetics & Germplasm Enhancement and Utilization, Ministry of Agriculture and Rural Affairs Key Laboratory of Biology and Germplasm Enhancement of Horticultural Crops in East China, College of Horticulture, Nanjing Agricultural University, Nanjing, Jiangsu, China; 2 Facility Horticulture Research Institute of Suqian, Suqian Research Institute of Nanjing Agricultural University, Suqian, Jiangsu, China

**Keywords:** Daylily, Quality, Rehydration, Temperature, Microstructure

## Abstract

Daylily is a unique cash crop in China which uses the unopened flower buds as food organs. Fresh daylily easily deteriorates and dried daylily is the main supply form in the market at present. The main goal of this work is to determine dried daylily water rehydration properties and sugar, lignin, cellulose, carotenoid, flavonoid and polyphenol retention properties under different temperatures and time periods. In this study, daylily was soaked at room temperature (25 °C), 50 °C and 70 °C for different durations. The results showed that after rehydration, the brightness index of daylily increased, while the redness and yellowness value decreased. The higher the water temperature, the faster the water absorption expansion rate and the higher the rehydration rate of the dried daylily, but the higher water temperature can cause the destruction of the microstructure of the daylily. The soluble sugars of dried daylily were easily lost in the rehydration process, the contents of lignin, cellulose and carotenoid were relatively stable. The contents of polyphenols and flavonoids decreased significantly with the increase of rehydration time. The rehydration of dried daylily can be carried out at room temperature, and it is recommended that the rehydration time take 0.5 h.

## Introduction

Daylily (*Hemerocallis citrina*) is a perennial herb of the *Hemerocallis* in the liliaceae, whose flower buds can be eaten as vegetables (Liu et al., 2020). Daylily is a unique cash crop in China, which has a long cultivation history and a wide planting range in China (Jia et al., 2024). For many years, the market value of daylily has remained at around 1 billion US dollars (Qing et al., 2021). Daylily is an excellent plant resource that has the concomitant function of both medicine and foodstuff. It contains various nutrients such as soluble sugars, ascorbic acid, flavonoids, dietary fiber, carotenoids, mineral elements, and polyphenols (Wang et al., 2024b). It is also rich in abundant anthraquinones, terpenoids, alkaloids and other bioactive substances, and has excellent performance in antioxidant, anti-tumor, antibacterial, anti-inflammatory, liver protection and so on (Liu et al., 2022; Ma et al., 2022; Ti et al., 2022). However, fresh daylily is difficult to preserve, and if not handled properly during consumption, it is prone to causing food poisoning after consumption. So, most of the daylily sold in the market are dried vegetables after processing (Qing et al., 2021; Chu et al., 2023). Therefore, how to ensure that dried daylily retain their excellent nutritional value during consumption has become a matter of great concern for consumers.

Dried vegetables are usually rehydrated before eating. Due to the wide variation of drying technologies, rehydration conditions and rehydration time, the rehydration effect of dried vegetables will be affected differently (Dong et al., 2019; Zhao et al., 2020; Chu et al., 2023). Temperature is one of the important factors affecting the rehydration of dried vegetables. Temperature will affect the changes of cell structure during rehydration, which will lead to the differences in appearance, color and rehydration of dried vegetables, and also affect the quality traits such as antioxidant substances in food (Dong et al., 2019; Zhao et al., 2020; Wang et al., 2023a). Previous studies have shown that the contents of soluble dietary fiber and pectin decreased with the increase of rehydration temperature, resulting in the destruction of interstitial and matrix. The higher the rehydration temperature, the greater the degree of cell wall destruction, and the stronger the membrane permeability, the more obvious the rehydration effect (Walawalkar, Poosarla & Shivshetty, 2023; Wang et al., 2023b). For soaking dried fungus, shiitake mushroom, and sliced bitter melon, the appropriate rehydration water temperature is 40∼55 °C (Yan et al., 2019; Zhang et al., 2022), which provides guidance for consumers’ consumption, while there are relatively few studies on the effects of rehydration conditions on the quality, texture and flavor of dried daylily.

Soluble sugar is soluble in biological cells and can be extracted by water and other polar solvents. It is one of the important components of vegetable quality. Water had a significant effect on soluble sugar content in plant cells (Gao et al., 2022). Cellulose is the main component of insoluble dietary fiber, and most vegetables and grains are rich in insoluble dietary fiber, such as beans, citrus fruits, barley, and carrots. Insoluble dietary fiber may improve gastrointestinal function by enhancing intestinal motility and microbial proliferation (Tian et al., 2024). Lignin is a complex phenylpropane-derived polymer that supports plants and resists stress. Plant lignin is filled in the cellulose skeleton, and higher lignin content will thicken plant cell walls, thus affecting the edible taste of vegetables to a certain extent (Liu, Luo & Zheng, 2018). Polyphenols are a general term for compounds with single or multiple phenolic groups in plants, which can be roughly divided into flavonoids, stilbenes, phenolic acids and lignans according to their structure. Plant polyphenols have antioxidant, antiviral, antibacterial and other biological activities, and are widely used in food, animal husbandry and aquatic products. Flavonoids are yellow secondary metabolites widely present in natural plants, with flavonoid (2-phenylchromone) as the parent nucleus, including flavonoids, rutin, hesperidin, *etc.*, which are water-soluble (She et al., 2022). Carotenoids are terpenoid secondary metabolites that exist in nature and are red, orange and yellow in color, mainly including lycopene, lutein, α-carotene and β-carotene. Both flavonoids and carotenoids have free radical scavenging and antioxidant effects, and play an important role in anti-cancer and anti-aging. The flavonoids and carotenoids in daylily also provide its color appearance and antioxidant nutritional value (Langi et al., 2018; Qing et al., 2021).

The main goal of this work is to determine dried daylily water rehydration properties and sugar, lignin, cellulose, carotenoid, flavonoid and polyphenol retention properties under different temperatures and time periods.

## Material & Methods

### Plant materials

Dried daylily from Changbai Mountain, Tonghua City, Jilin Province was used as experimental material. The unblooming buds of daylily were harvested manually in summer. Then, following the method of Wang et al. (2023c), the daylily was placed in a sealed transparent environment and exposed to sunlight (6 h) for fixation. Finally, the daylily was dried by being left to air-dry for 14 h in a natural environment with an average temperature of 25 °C and an average humidity of 60%, so that their moisture content ranged from 0.1∼0.2 g/g DW (dry weight). The flower buds of daylily can be roughly regarded as cylindrical vegetables, with a diameter of approximately 5.2 ± 0.3 mm, and a length of about 72.9 ± 4.9 mm. The initial average weight of dried daylily was 0.45 g.

### Rehydration of dried daylily

In order to investigate the influence of different temperature on the rehydration of daylily, the evenly sized dried daylily was divided into three groups (each group containing 100 daylily sticks) and put into three 2 L glass beakers. Three groups of dried daylilies were soaked in tap water at room temperature (25 °C), 50 °C and 70 ° C, respectively. During the process, the daylily buds were fully submerged in the water all along, but no stirring was performed. The morphology and color changes of daylily buds were recorded before and after soaking for 0.5 h, 2 h and 4 h at each time point, respectively.

### Determination of rehydration ratio and average rehydration ratio rate

The weight of individual buds was determined using an analytical balance. A total of 20 daylily buds were selected from each group and weighed. The weight difference of rehydrated daylilies under different temperature at different time was recorded to calculate the rehydration ratio and average rehydration ratio rate. The calculation of the rehydration ratio followed the method proposed by Zhao et al. (2020). The method for calculating the average rehydration rate was based on the drying rate calculation formula proposed by Ding et al. (2012), which was as follows:

Average rehydration ratio rate (V_f_) = m_1_-m_0_/t.

m_1_ represents the weight of the rehydrated daylily sample (g), m_0_ represents the initial weight of the sample (g), and t is the rehydration time (h).

### Microstructure observation

In order to explore the effect of rehydration under different temperatures on the anatomical structure of daylily, the cross sections of daylily bud under different treatments were stained and sliced according to Jia et al. (2023). First, the sampled daylily buds were fixed with FAA (formaldehyde-acetic acid-ethanol) fixation solution, and then paraffin sections were prepared by embedding in the tissues. Secondly, the prepared paraffin sections were placed in xylene and anhydrous ethanol for dewaxing and rehydration treatment, and then placed in the saffranine dye solution at room temperature for 1∼2 h. The excess dye solution was washed with distilled water. The slices were then placed in 50%, 70% and 80% ethanol in turn for decolorization treatment, and then dyed with solid green dye solution for a second time for 30∼60 s. After washing off the excess dye solution with ethanol, neutral gum was used for sealing. Slices were observed by fluorescence microscope Olympus BX53 in the State Key Laboratory of Crop Genetics & Germplasm Enhancement and Utilization. After staining with saffranine solid green, the fibrotic tissue was dyed green, and the lignified tissue was red. The spontaneous fluorescence of lignin was observed under a wavelength of 405 nm (Pérez-de Lis et al., 2024).

### Determination of color

After soaking the daylily in water for different periods of time, the water stains on the surface were wiped off with absorbent paper. The color difference value of the daylily was measured using the colorimeter CHROMA METER CR-400. The values of L, a, and b were recorded. L is the lightness index. *L* = 0 indicates black, and *L* = 100 indicates white. The value of a represents the redness, and the larger the value, the closer it is to pure red. The value of b represents the yellowness, and the larger the value, the closer it is to pure yellow (Zhou et al., 2022).

### Determination of soluble sugar content

Anthrone method was used to detect the soluble sugar content of daylily before and after rehydration (Yue et al., 2022). The sample was ground in a mortar with liquid nitrogen, and then freeze-dried. About 0.1 g of the dried sample was weighed. Distilled water was used to extract soluble sugars from the samples in a boiling water bath. After cooling and centrifuging at 8,000 *g* for 10 min, the supernatant was taken and volumed with distilled water to 10 mL. Anthrone reagent was added to the above obtained solution. After a water bath of 95 °C for 10 min, the absorbance was detected at 620 nm wavelength. The soluble sugar content was calculated according to the standard curve. Different concentration gradients of glucose were used as the standard. Three biological replicates were set up.

### Determination of flavonoid content

The determination of flavonoid content was performed according to the method of Wang et al. (2022). Approximately 0.2 g dry weight daylily sample was used for flavonoid determination. The sample was soaked with 60% ethanol in a 50 °C water bath for 2 h. A concentration of 5% NaNO_2_ was added to the extraction solution and shaked well. After standing, 10% Al(NO_3_)_3_ was added. Terminating agent 1 mol/L NaOH was added after standing again. After diluting with 70% ethanol to volume, the absorption was determined at 500 nm. Rutin standard solution was used for the standard curve and flavonoid content was calculated.

### Determination of lignin content

The content of lignin in daylily was determined by thioglycolic acid method (Dampanaboina, Yuan & Mendu, 2021). The sample was ground with liquid nitrogen and mixed in anhydrous ethanol. After centrifuging at 14,000 rpm for 20 min, the precipitate was collected and air-dried at room temperature overnight. About 10 mg of dry sample were weighed and placed in 2 M HCl with thioglycolic acid. The mixture was heated at 100 °C for 8 h and cooled on ice. The precipitate was collected by centrifugation and washed with deionized water, then dissolved with 1 M NaOH for 18 h. The resting liquid was centrifuged at 14,000 rpm for 20 min to collect the supernatant. After adding one mL of concentrated HCl at 4 °C for 6 h, the lignin precipitate was collected and dissolved with 1 M NaOH for determination. The detection wavelength was 280 nm, and 1 M NaOH was used as control. Three biological replicates were set up.

### Determination of cellulose

The determination of cellulose was performed by anthrone-sulfuric acid method according to previous methods (Xue et al., 2023). The samples were set up with three biological replicates.

### Extraction and determination of carotenoids

The carotenoids in the sample were extracted using acetone. The total carotenoid content was calculated by measuring the absorbance at 663 and 646 nm (Islam et al., 2016).

### Determination of polyphenol content

Polyphenol content was determined by folin phenol method (Attard, 2013). Daylily sample was ground into powder in liquid nitrogen, and homogenized under ice bath after adding 10 mL 80% ethanol. The supernatant obtained after centrifugation was used as crude polyphenol extraction. The reaction solution consisted of the extraction solution, 0.5 M folinol solution and 0.5 M Na_2_CO_3_, which was shielded from light for 1 h. The absorption was determined at 750 nm and gallic acid was used as standard. Three biological replicates were set up.

### Data analysis

The data obtained were organized, analyzed and graphed by Microsoft Office Excel. Duncan’s multiple comparative analysis was performed using SPSS 25.0 (*P* < 0.05; IBM Corp, Armonk, NY, USA), data was expressed as the mean ± standard deviation of three biological replicates.

## Results

### Changes of morphology and color of daylily during rehydration at different temperatures

**Figure 1:**
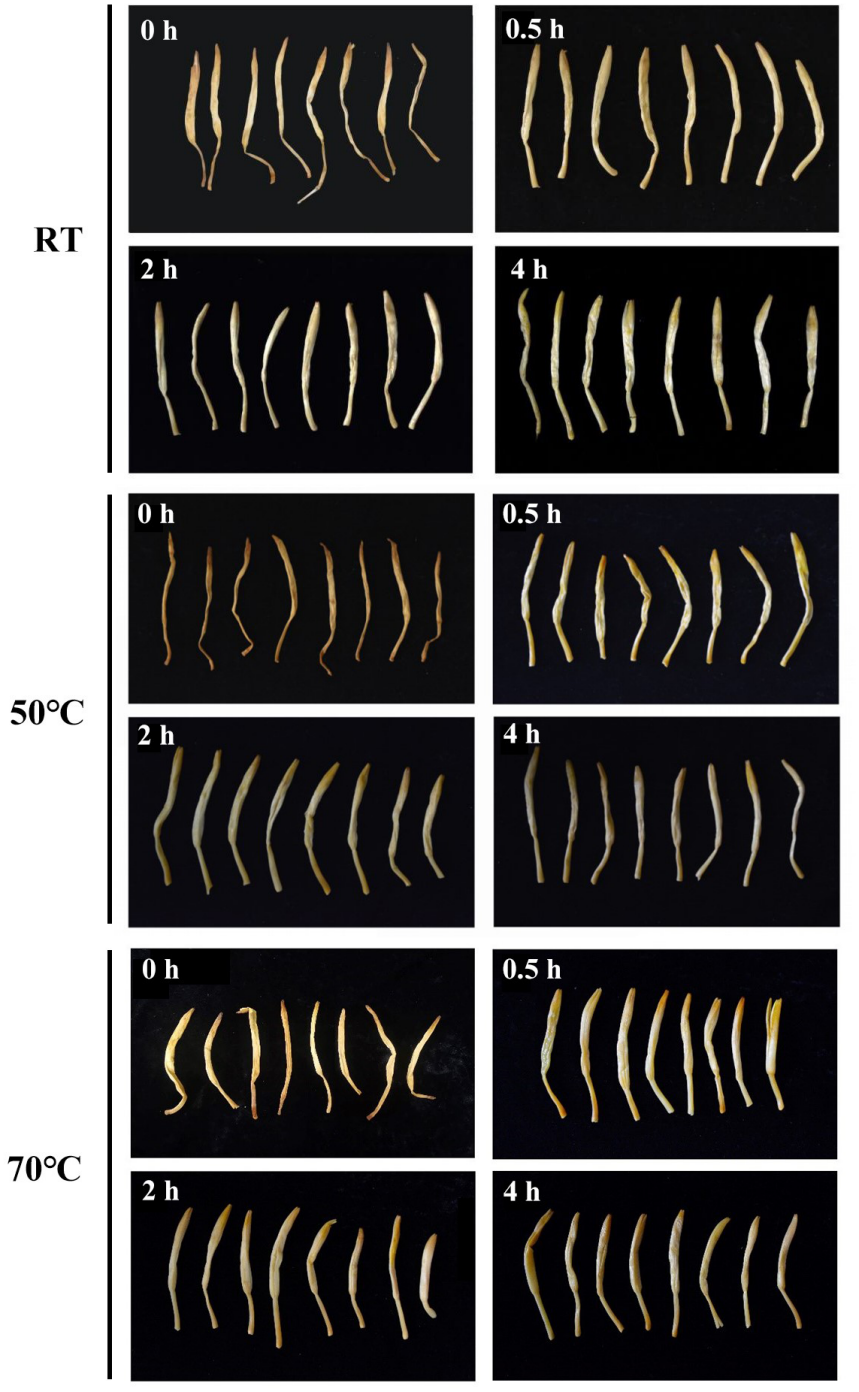
Morphology of daylily during rehydration at different temperatures.

The morphology of daylily during the rehydration process was shown in Fig. 1. The color of the flower buds of daylily changed from dark yellow to light yellow, the buds expanded after absorbing water. Comparing the rehydration processes at different water temperatures, it was found that daylily expanded faster when soaked in 50 °C and 70 °C water. The flower buds of daylily were full and expanded when soaked for 0.5 h at 70 °C water temperature.

The statistical values of the color of daylily samples after rehydration at different temperatures for different times were shown in Table 1. During the rehydration process, the brightness (L value) of daylily increased, while the redness (a value) and yellowness (b value) indexes decreased. After rehydrating at different temperatures for 4 h, L, a and b values of daylily changed from the initial 48.40, 6.62 and 31.17 to 66.87, 3.61, 27.79 (RT), 68.43, 3.10, 28.58 (water temperature 50 °C), and 65.25, 2.29, 25.41 (water temperature 70 °C), respectively. The results showed that the redness and yellowness values of daylily color were the lowest after soaking at 70 °C for 4 h.

**Table 1:** Statistical data of color of daylily under different rehydration temperatures.

Time	Index	RT			50 °C			70 °C		
		Value	SD	CV/%	Value	SD	CV/%	Value	SD	CV/%
0 h	L	48.40	7.26	15.00	48.40	7.26	15.00	48.40	7.26	15.00
	a	6.62	1.44	21.75	6.62	1.44	21.75	6.62	1.44	21.75
	b	31.17	4.68	15.01	31.17	4.68	15.01	31.17	4.68	15.01
0.5 h	L	61.83	4.46	7.21	65.74	2.63	4.00	64.10	3.24	5.05
	a	5.50	1.26	22.90	4.07	0.90	0.22	3.96	0.78	19.70
	b	29.38	2.30	7.80	31.33	2.61	8.33	29.59	5.75	19.43
2 h	L	66.02	2.88	4.36	64.81	4.88	7.53	61.74	5.74	9.30
	a	3.27	1.02	0.31	3.29	0.71	21.58	2.94	1.00	34.01
	b	30.23	5.77	19.08	26.38	1.52	5.76	23.76	6.61	27.82
4 h	L	66.87	3.46	5.17	68.43	3.73	5.45	65.25	3.77	5.78
	a	3.61	0.89	24.65	3.10	0.93	30.00	2.29	0.84	36.68
	b	27.79	3.10	11.15	28.58	3.05	10.67	25.41	2.07	8.15

**Notes.**

SD Standard deviation CV Coefficient of variation

### Changes of microstructure of daylily during rehydration at different temperatures

**Figure 2:**
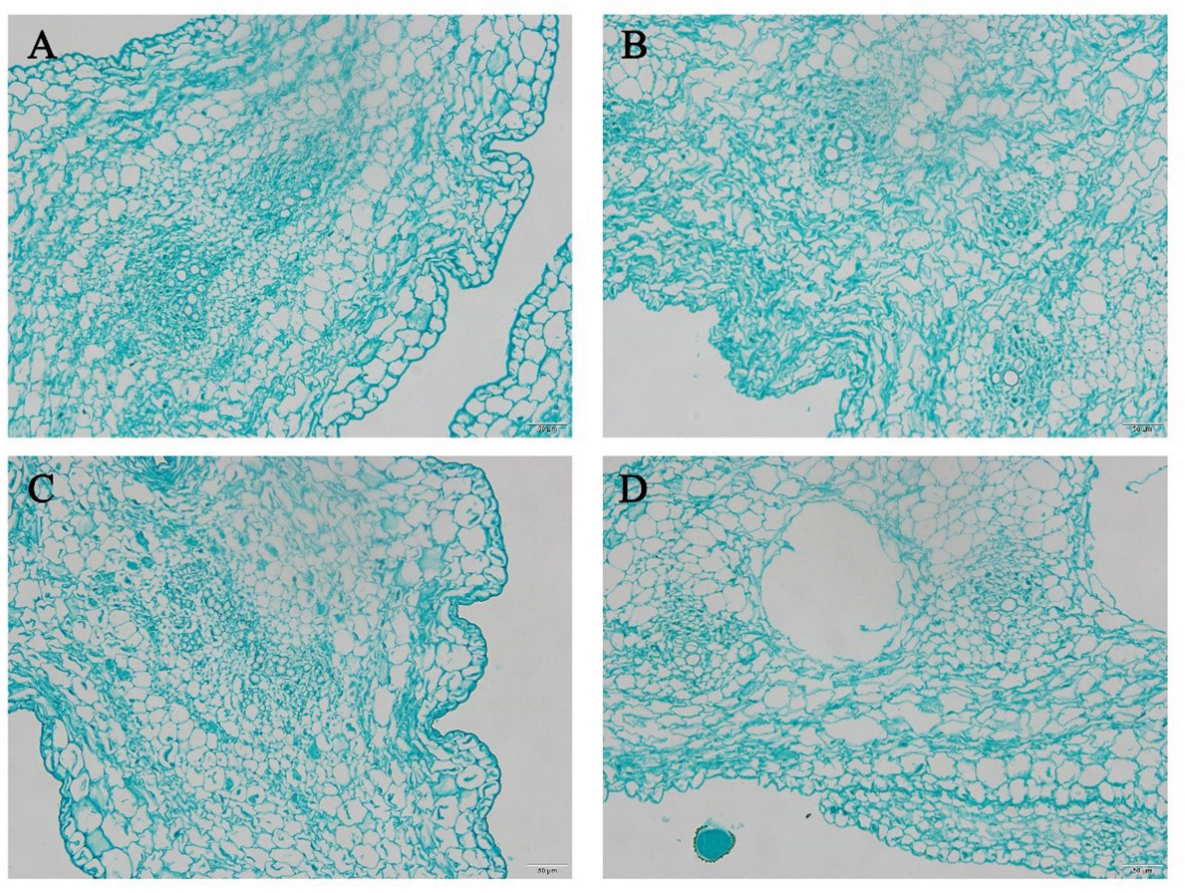
Microstructure of daylily before and after rehydration at different temperatures. (A) Cell structure of dried daylily before rehydration. (B) Cell structure of dried daylily after rehydration for 4 h at room temperature. (C) Cell structure of dried daylily after rehydration for 4 h at 50 °C. (D) Cell structure of dried daylily after rehydration for 4 h at 70 °C.

The microstructures of the dried daylily before and after soaking at different temperatures for 4 h were observed and compared in slices (Fig. 2). After rehydrating the dried daylily for 4 h, the water content in the samples was restored and the cell structures gradually expanded. Compared with the rehydration treatment carried out at room temperature (Fig. 2A), the water soaking after heating caused minor damage to the cells. Figures 2B and 2C showed the microstructure of the daylily during rehydration at room temperature and at 50 °C. Some cell structures showed slight damage, and the microstructure of daylily was also somewhat disrupted. With under water soaking at 70 °C (Fig. 2D), this phenomenon was more obvious.

### Effect of different temperatures on rehydration of dried daylily

**Figure 3:**
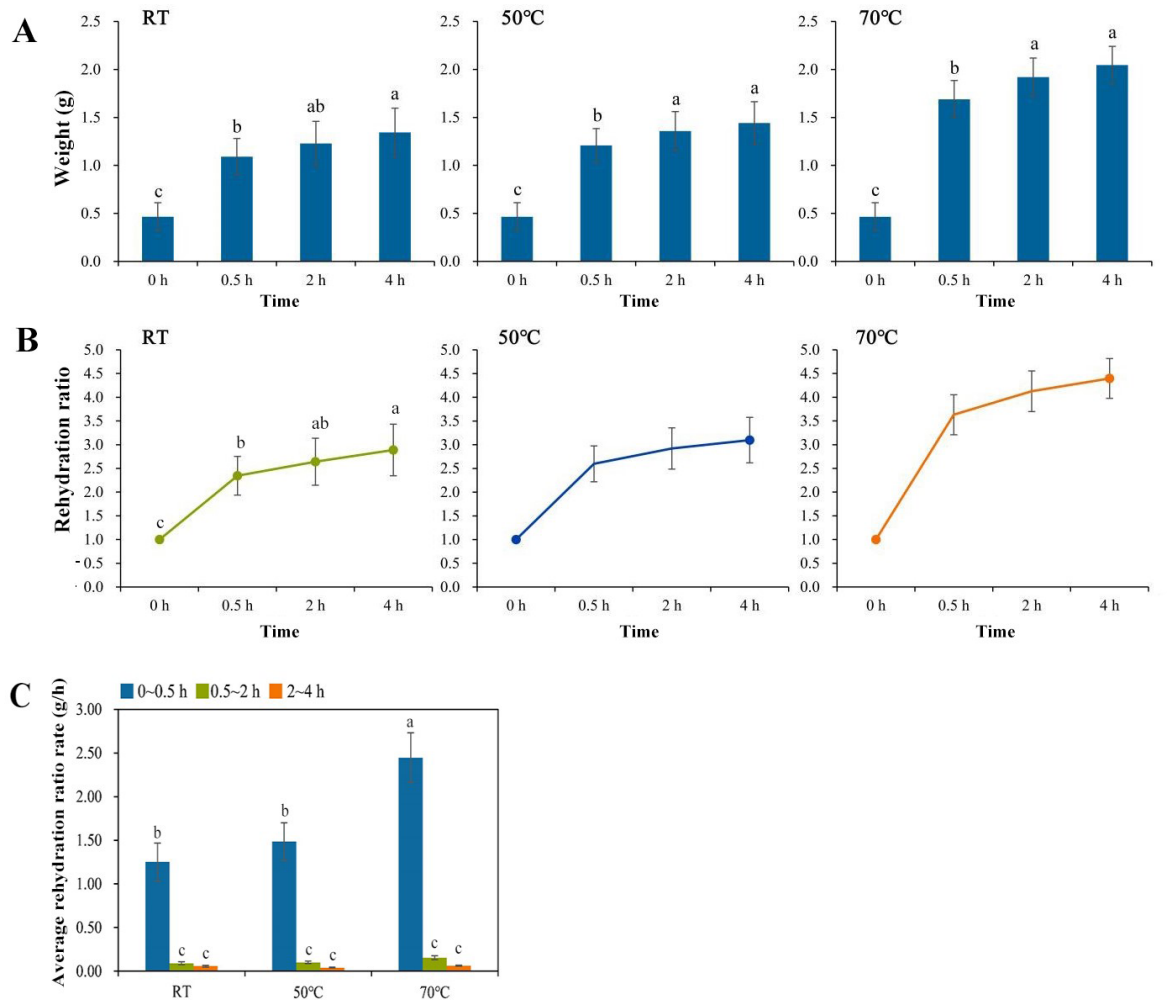
Rehydration of daylily under different rehydration conditions. (A) Weight change of daylily under different rehydration conditions. (B) Rehydration ratio of daylily under different rehydration conditions. (C) Average rehydration rate of daylily under different rehydration conditions. Different lowercase letters indicate significant differences (*P* < 0.05).

The weight and volume of dried daylily increased during the rehydration process. The recorded weight data of daylily at different time points under different water temperatures showed that the weight of daylily increased rapidly after water immersion at room temperature, 50 °C and 70 °C, and the weight increased slowly after rehydration for 0.5 h (Fig. 3A). After rehydration for 0.5 h, the average weight of single daylily bud was 1.09, 1.21 and 1.69 g, respectively. After rehydration for 2 h, the average weight of single daylily was 1.23, 1.36 and 1.92 g, respectively. After rehydration for 4 h, the average weight of single daylily tended to be stable, reaching 1.34, 1.44 and 2.05 g, respectively. Compared with room temperature soaking, the weight of daylily was higher for 0.5 h, 2 h and 4 h after soaking in warm water at 50 °C and 70 °C, and the weight of daylily was increased most significantly under soaking in warm water at 70 °C.

The rehydration ratio of daylily after rehydration in water at different temperatures for different time was calculated. The results (Fig. 3B) showed that after soaking at room temperature for 0.5 h, 2 h and 4 h, the rehydration ratio of daylily was 1.35, 1.64 and 1.89, respectively. After soaking in warm water at 50 °C for 0.5 h, 2 h and 4 h, the rehydration ratios of daylily were 1.57, 1.92 and 2.10, respectively. After soaking in warm water at 70 °C for 0.5 h, 2 h and 4 h, the rehydration ratios of daylily were 2.63, 3.13 and 3.40, respectively.

With the increase of rehydration time, the average rehydration rate of dried daylily gradually decreased (Fig. 3C). The average rehydration ratio rate of daylily was significantly different under different rehydration conditions. At first 0.5 h after rehydration, the average specific rehydration rate of daylily soaked at room temperature, 50 °C and 70  °C were 1.252, 1.486 and 2.448 g/h, respectively. After 0.5 h, the water temperature gradually decreased, the temperature difference decreased, and the difference of average rehydration rate under different water temperatures also narrowed.

### Changes of fiber of daylily during rehydration at different temperatures

**Figure 4:**
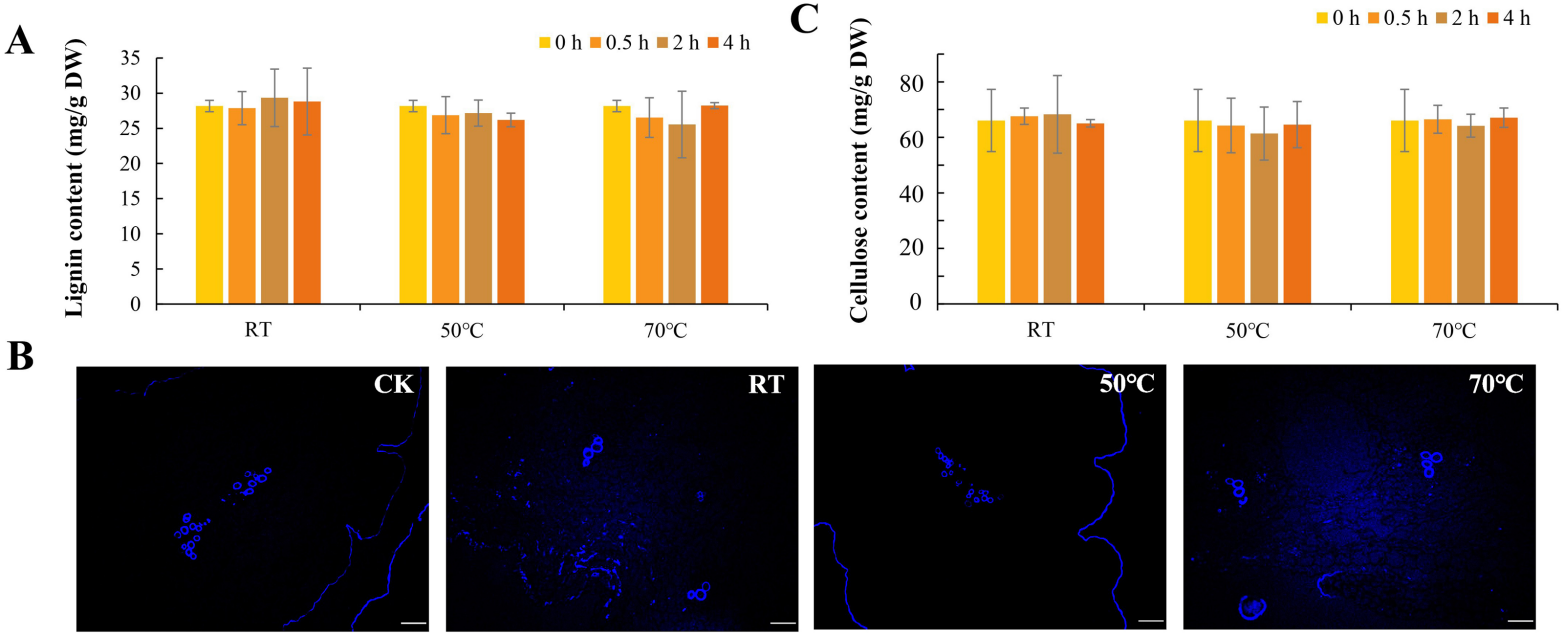
Fiber of daylily under different rehydration conditions. (A) Lignin content of daylily under different rehydration conditions. (B) Lignin autofluorescence of daylily under different rehydration temperature for 4 h. (C) Cellulose content of daylily under different rehydration conditions.

Fiber content affects the taste of daylily to some extent. Thioglycolic acid method was used to detect the lignin content in daylily after soaking in water at different temperatures for 0 h, 0.5 h, 2 h and 4 h (Fig. 4A). The results showed that there was no significant difference in lignin content in daylily buds before and after soaking at room temperature or at higher water temperature, and the lignin content remained relatively high stability, ranging from 25.54 to 29.34 mg/g DW, indicating that the lignin content in daylily was not affected by rehydration conditions such as water temperature and soaking time.

The microstructures of the flower buds of daylily were observed by microscope at room temperature, 50 °C and 70  °C under tap water (Fig. 4B). The lignin showed a spontaneous fluorescence phenomenon under UV excitation. As shown in the figure, lignin was mainly distributed in the exodermis and the xylem region of the cross-section. Rehydration treatment with water at different temperatures has no significant effect on the lignin accumulation of daylily.

Cellulose is a kind of complex carbohydrate that cannot be digested by the human body and is an important part of dietary fiber. In order to investigate the effect of different rehydration temperature on cellulose content in daylily, the change of cellulose content in daylily soaked in tap water at room temperature, 50 °C and 70 °C was detected. As shown in Fig. 4C, the cellulose content of the flower buds of daylily ranged from 61.4 to 68.3 mg/g DW, and the cellulose content of daylily did not change significantly during the rehydration process, indicating that the cellulose content of daylily was relatively stable and was not affected by rehydration conditions such as water temperature and time.

### Changes of total flavonoids of daylily during rehydration at different temperatures

**Figure 5:**
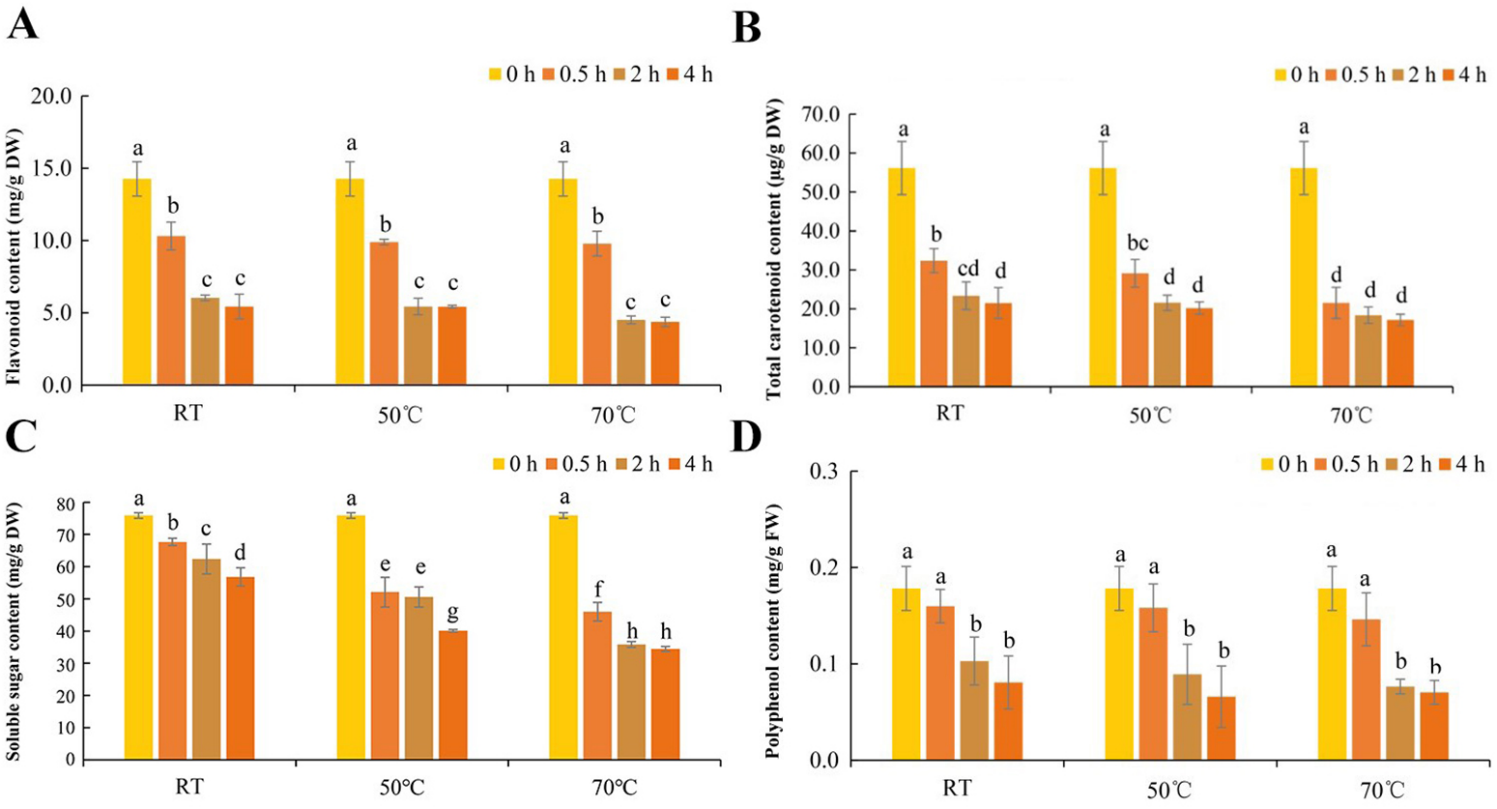
Changes in nutrient content of daylily under different rehydration conditions. (A) Total flavonoid content of daylily under different rehydration conditions. (B) Total carotenoid content of daylily under different rehydration conditions. (C) Soluble sugar content of daylily under different rehydration conditions. (D) Polyphenol content of daylily under different rehydration conditions. Different lowercase letters indicate significant differences (*P* < 0.05).

Flavonoid content is an important index of daylily quality which is closely related to the antioxidant capacity of daylily. The determination of flavonoid content after rehydration treatment (Fig. 5A) showed that rehydration treatment caused the decrease of flavonoid content in daylily. The main factor affecting the loss of flavonoid content was rehydration time. Rehydration temperature had little effect on the change of flavonoid content. The highest flavonoid content was 14 mg/g DW in dried daylily samples without rehydration. Compared with dried daylily before rehydration, flavonoid content decreased significantly after rehydration for 0.5 h, which was 10 mg/g DW. After rehydration for 2 h, flavonoid content continued to halve to five mg/g DW, and the flavonoid content at 4 h was similar to that at 2 h. The above results showed that the flavonoids in daylily reached the maximum loss at 2 h after rehydration, and the loss rate was not affected by different rehydration temperatures.

### Changes of total carotenoids of daylily during rehydration at different temperatures

The contents of total carotenoids in dried daylily after rehydration under different rehydration conditions were determined (Fig. 5B). The results showed that the total carotenoid content decreased significantly within 2 h of rehydration. After rehydration for 0.5 h, the content of total carotenoids decreased to 1/3 to 1/2 of that before rehydration. The lowest total carotenoid content in the daylily samples was 17 µg/g DW which was soaked at 70 °C for 4 h.

### Changes of soluble sugar of daylily during rehydration at different temperatures

After rehydrating dried daylily with water at different temperatures, the soluble sugar content showed a step-down trend with the increase of rehydration temperature. At the same rehydration temperature, soluble sugar content decreased with the increase of rehydration time (Fig. 5C). The soluble sugar content in the samples before rehydration was approximately 75 mg/g DW, and the lowest soluble sugar content was 34 mg/g DW in the samples incubated at 70 °C for 4 h. The content of soluble sugar in the unsoaked sample was 2.2 times that of the sample after soaking at 70 °C for 4 h. The results indicated that high temperature and long rehydration conditions would accelerate the loss of soluble sugar in daylily.

### Changes of polyphenol of daylily during rehydration at different temperatures

Compared with the control, the polyphenol content of daylily after soaking in different rehydration temperatures for different time decreased in different degrees (Fig. 5D). Before rehydration, the polyphenol content of dried daylily was about 0.178 mg/g FW. With the extension of rehydration time, polyphenol content in daylily samples decreased gradually, and the difference was significant at 2 h of rehydration, which decreased to 0.43 to 0.57 times of untreated. After rehydration for 2 h, the polyphenol content in the sample continued to decrease, but the decline rate slowed down. The difference was not significant under different water temperatures. When rehydration for 4 h, the polyphenol content of rehydrated daylily under different water temperatures was 0.081, 0.066 and 0.070 mg/g FW, respectively.

## Discussion

As a perennial herb of the Liliaceae, daylily is different from most vegetables with roots, stems and leaves as edible organs, it mainly uses unopened flower buds as edible organs (Liu et al., 2020). Daylily contains rich bioactive substances, data have shown that daylily contains colchicine, eating fresh daylily will cause poisoning. In 2021, the genome of daylily was analyzed and published, which proved that daylily does not contain colchicine and its precursor compounds based on metabolome data, and clarified the biological basis of its colchicine-free from the genomic level (Qing et al., 2021). Despite this, there are still reports of people being poisoned after eating fresh daylily. Therefore, when harvesting daylily, it is necessary to steam, dehydrate, dry and preserve it. The drying treatment of daylily can not only remove the toxic substances, but also extend the storage time of daylily to facilitate transportation and sales (Jia et al., 2024).

The quality of dry products is related to the methods and conditions of the drying process as well as the rehydration process. Dry products are usually rehydrated before food processing, and the temperature during rehydration is an important factor affecting the rehydration efficiency. Some dry products can be rehydrated in cold water, while some need to be soaked in warm or hot water to completely bubble and expand (Chu et al., 2023; Wang et al., 2023a; Wang et al., 2023b). Generally speaking, the water reabsorbing capacity of dry products with higher water temperature is faster, but higher water temperature may cause the loss of nutrients in dry products or the taste decline. Only when the dried products are processed using the most optimal drying methods and the water temperature during the rehydration process is properly controlled, can dried products be completely soaked and restore their original freshness (Zielinska et al., 2020; Chu et al., 2023).

The current research mainly focuses on the drying environment of daylily, and for different production purposes, there have been studies that have provided relatively clear suggestions in the drying process, such as freeze-drying and microwave freeze drying are applicable to daylily powder with high nutritional value, while hot-air drying and heat pump drying are suitable for large-scale production and affordable dried daylily products (Chu et al., 2023). However, there are relatively few comparisons regarding the rehydration conditions of daylily. The methods that can be referred to include rehydration at 30 °C for 2 h and soaking at room temperature for 40 min (Wang et al., 2015; Chu et al., 2023). Agaric, vermicelli and kelp are always soaked in cold water (Zhang et al., 2022). As for lentinusedodes, warm water is usually used to make it easier for absorbing water, and the guanylate can fully decompose and give off umami flavor (Qiu et al., 2021). The dried bamboo shoots and lilies should be soaked in hot water, which can be directly poured into boiling water and braised for soaking treatment (Chongtham et al., 2022; Zhang et al., 2023).

In this study, room temperature, 50 °C and 70 °C water were utilized to rehydrate dried daylily. The results showed that the daylily can be basically soaked after 0.5 h of rehydration with 70 °C water, and the dried daylily has rapidly absorbed, expanded and softened, which can be used for subsequent cooking treatment. The higher the temperature of water used, the higher the rehydration efficiency of dried daylily. After rehydrating the daylily with water of different temperatures, the brightness of daylily increased, while its yellowness and redness decreased. This indicates that the brightness has improved, but the saturation has slightly decreased, suggesting that there might be a loss of water-soluble pigments during the rehydration process. Based on the moisture content of fresh daylily provided in the study by Ding et al. (2012), which was 10.13 g H_2_O/g DW, the initial DW of daylily in this study was approximately 0.4 g. If the dried daylily would be totally hydrated, the final weight would be approximately 4.4 g. To obtain the rehydration equivalent to a fresh daylily, the rehydration ratio can be calculated to reach approximately 9.78. Although the rehydration ratio of dried daylily was the highest following 2 h of rehydration at 70 °C, it only reached 4.10. This finding indicates that the rehydration process of dried daylily is irreversible, as their moisture content cannot be restored to the level of fresh daylily through rehydration.

According to previous research, the higher the temperature of the soaking water may also cause the loss of internal nutrients (Dong et al., 2019; Zhao et al., 2020). In this study, the contents of soluble sugar, flavonoids, lignin, cellulose, carotenoids and total phenol in daylily were measured under different rehydration temperature and time. The results showed that the soluble sugar content of daylily was significantly lost during rehydration process, and the loss of soluble sugar can be aggravated with the increase of rehydration temperature and rehydration time. It is speculated that soluble substances similar to soluble sugar properties, such as soluble protein, may also appear similar regular loss after rehydration. During the dehydration and rehydration process of vegetables, the changes in lignin and cellulose content may be related to the permeability of the cell membrane and the reformation of the pectin-cellulose network in the cell wall (Wang et al., 2024a). There was no significant difference in lignin content between the mushrooms treated with hot air and freeze-drying after rehydration, while the cellulose content would increase under hot air drying (Qiu et al., 2022). As for daylily, after rehydration treatment under different temperature conditions, there was no significant difference in the content of cellulose and lignin. This indicates that the drying method may have a more significant impact on cellulose and lignin. Lignin has strong resistance to degradation and is usually not affected by the rehydration temperature (Wang et al., 2024a). Similarly, the rehydration did not cause significant changes in the content of cellulose in daylily. This result might be related to the insolubility and thermal stability of cellulose.

As an important secondary metabolite of plants, flavonoids play an important role in plant growth, development and stress resistance. In the detection of flavonoids in flower buds and roots of daylilies, it was found that flavonoids in daylilies included flavonol derivatives, isoflavone derivatives, chalcone derivatives and dihydroflavonoids, among which quercetin glycosides had the highest content (Lv & Guo, 2023). Compared with the reported total flavonoid content, the flavonoid content detected in this study was lower than that reported, which may be due to the loss of a large amount of flavonoid in the early processing and drying of daylily. The flavonoid content can maintain a certain stability at higher water temperature, but will be significantly lost after 2 h rehydration. Carotenoids, which include carotenes and xanthophylls, are the main source of bright colors in various fruits and vegetables (Langi et al., 2018). The results of the detection of carotenoid content in daylily showed that the carotenoid content in daylily was low, the total carotenoid content of dried daylily before rehydration was 56 ug/g DW. The total carotenoid content decreased to about half of that before rehydration at 0.5 h after rehydration. Although carotenoids are fat-soluble compounds, the results suggest that the less stable carotenoids may be degraded during rehydration. In this experiment, the total polyphenol content was also determined under different rehydration conditions. The result was similar to the change trend of flavonoid content. Temperature had little effect on the total phenol content, and the increase of rehydration time leaded to significant loss of polyphenols.

## Conclusions

In this study, dried daylily was soaked at room temperature (25 °C), 50 °C and 70 °C for different durations. The morphology, color and rehydration efficiency of dried daylily during the soaking process were compared, and the contents of some nutrients were further evaluated. The results indicated that higher water temperature is conducive to rapid water absorption and expansion of daylily flower buds. However, excessively higher water temperatures during rehydration can cause the cell structure to rupture and cause slight damage to the microstructure of daylily. Therefore, it is recommended to use room temperature water for rehydration, and the quality of daylily can be maintained when it is rehydrated at room temperature for 0.5 h.

##  Supplemental Information

10.7717/peerj.20408/supp-1 Supplemental Information 1 Raw data for all figures

## Additional Information and Declarations

### Data Availability

The following information was supplied regarding data availability: The raw measurements are available in the Supplementary File.
